# A novel polyester–drug nanoconjugate with subtype selectivity for high efficacy against PSMA-positive prostate cancer

**DOI:** 10.1093/rb/rbag072

**Published:** 2026-04-11

**Authors:** Shiwei Guo, Xiyan Xu, Guobing Li, Yongfeng Yang, Zhihui Yan, Xulin Wang, Pei Jing, Yong Zhou, Man Jia, Yuanfu Wang, Yan Dai, Siping Wei, Ronghao Wang, Bo Cheng

**Affiliations:** Department of Pharmacy, The Affiliated Hospital of Southwest Medical University, Luzhou, Sichuan 646000, China; Department of Histology and Embryology, Southwest Medical University, Luzhou, Sichuan 646000, China; Department of Biochemistry and Molecular Biology, School of Basic Medical Sciences, Southwest Medical University, Luzhou, Sichuan 646000, China; Department of Pharmacy, The Affiliated Hospital of Southwest Medical University, Luzhou, Sichuan 646000, China; Department of Urology, The Affiliated Hospital of Southwest Medical University, Luzhou, Sichuan 646000, China; Department of Biochemistry and Molecular Biology, School of Basic Medical Sciences, Southwest Medical University, Luzhou, Sichuan 646000, China; Department of Pharmacy, The Affiliated Hospital of Southwest Medical University, Luzhou, Sichuan 646000, China; Department of Biochemistry and Molecular Biology, School of Basic Medical Sciences, Southwest Medical University, Luzhou, Sichuan 646000, China; Department of Biochemistry and Molecular Biology, School of Basic Medical Sciences, Southwest Medical University, Luzhou, Sichuan 646000, China; Department of Urology, The Affiliated Hospital of Southwest Medical University, Luzhou, Sichuan 646000, China; Department of Pharmacy, The Affiliated Hospital of Southwest Medical University, Luzhou, Sichuan 646000, China; Department of Pharmacy, The Affiliated Hospital of Southwest Medical University, Luzhou, Sichuan 646000, China; Department of Biochemistry and Molecular Biology, School of Basic Medical Sciences, Southwest Medical University, Luzhou, Sichuan 646000, China; Department of Urology, The Affiliated Hospital of Southwest Medical University, Luzhou, Sichuan 646000, China

**Keywords:** prostate cancer, prostate-specific membrane antigen, docetaxel, polyester, subtype selectivity

## Abstract

Prostate-specific membrane antigen (PSMA) positive prostate cancer (PCa) accounts for 80–90% cases. Docetaxel (DTX) is a first-line chemotherapy drug for metastatic castration resistant prostate cancer (mCRPC) and has exhibited promising efficacy. However, DTX usually causes severe side effects. To address this issue, we utilized short-chain poly(ethylene glycol) (PEG) as a monomer to prepare a water-soluble polyester, which was further modified by a PSMA ligand, 2-[3-[5-amino-1-carboxypentyl]-ureido]-pentanedioic acid (DCL) and covalently linked to DTX via a disulfide bond, resulting in a novel PSMA–targeting polyester–drug conjugate (PET–DCL–DTX). Here, the introduction of short-chain PEG into the polyester skeleton could enhance its hydrophilicity and drug-loading capacity. This conjugate is amphiphilic and forms a nanostructure via self-assembly in aqueous solution, allowing it to passively accumulate in tumor tissues via the enhanced permeability and retention (EPR) effect. Particularly, due to the specific recognition of DCL for PSMA, this nanoconjugate exerts a profound inhibitory effect on PSMA-positive PCa cells. After efficiently entering the cells, this nanoconjugate undergoes sensitive cleavage of the disulfide bond owing to the reductive molecules such as glutathione and releases DTX to suppress the PSMA-positive PCa development. Moreover, an improved safety of this nanoconjugate was observed *in vivo* compared to DTX alone.

## Graphical Abstract

**Graphical Abstract:**
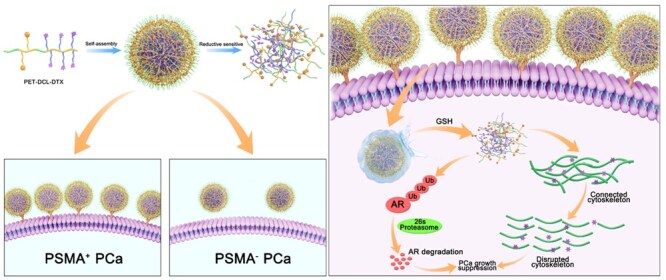

## Introduction

Prostate cancer (PCa) is the most common urological malignancy among men, which causes an estimated 375 000 deaths worldwide annually [1]. Advances in the molecular biology of PCa reveal that the prostate-specific membrane antigen (PSMA) is a specific biomarker of PCa owing to its high expression in prostate carcinoma compared to prostate tissues [2–4]. PCa can be roughly classified into three subtypes [5, 6]: AR-dependent PCa, cancer stem cell like PCa, and neuroendocrine like PCa. Generally, androgen-dependent PCa accounts for 80–90% of cases and is PSMA positive, which facilitates the development of PSMA-based diagnostic or therapeutic tools in the management of this type of disease [2, 7]. For example, Indium In 111 capromab pendetide or ProstaScint^®^ scan, a radiolabeled PSMA antibody (mAb 7E11), has been approved as a diagnostic approach to visualize metastatic PCa [8], which overrides the current computerized tomography scan or magnetic resonance imaging in the context of accuracy. In addition, the therapeutic value of PSMA has been observed in a clinical trial (NCT04227275), in which engineered PSMA specific chimeric antigen receptor T cells were tested in advanced PCa patients [9]. Moreover, some PSMA-based delivery systems have been reported in recent years for the treatment and diagnosis of PCa [10–12].

It is well known that the androgen receptor (AR) plays a key role in PCa carcinogenesis and progression, leading to the development of androgen deprivation therapy (ADT) as the golden treatment for PCa [13–16]. Nevertheless, the therapeutic duration of ADT only lasts 2–3 years as PCa progresses to a castration resistant state [17, 18]. Chemotherapy is commonly utilized in the management of castration resistant prostate cancer (CRPC) owing to its broad application and cost-effectiveness [19, 20]. Docetaxel (DTX), an anti-microtubule chemotherapy drug, has been approved by the Food and Drug Administration (FDA) for the treatment of metastatic CRPC or for managing castration sensitive PCa in combination with ADT [21, 22]. Clinical evidence has demonstrated that DTX prolongs patients’ survival by approximately 1 year [22]. However, severe side effects associated with DTX, such as fatigue, diarrhea and anemia, limit its clinical application [23, 24], which may be attributable to DTX’s lack of tumor targeting and the unexpected distribution to other tissues *in vivo*. Moreover, DTX faces challenges including poor solubility in water and suboptimal pharmacokinetic properties [25–27].

With regard to these issues, the use of versatile vectors to deliver small molecules is an effective strategy, and these carefully designed drug delivery systems (DDSs) can self-assemble into various nanomorphologies in the water environment [28–30]. Well-designed polymer–drug conjugates are a distinct class of nanoscale DDSs enabling the stealthy entry of drug molecules into the circulation system. These conjugates release cargoes to exert their intended functions in response to either internal or external stimuli when targeted at specific focal sites [31, 32]. Biodegradability is a core safety index for polymers used in DDSs. In this regard, polyesters have become promising candidates because they can be hydrolyzed by ubiquitous carboxylesterase *in vivo* [33]. However, the intrinsic hydrophobic property of polyesters significantly restricts their applications. Therefore, the polyesters can be hydrophilically modified for the improved water solubility. However, the introduction of additional hydrophilic components occupies a large number of functional groups and further increases the molecular weight of the carrier, ultimately leading to a dramatic decline in the drug-loading capacity [34].

**Figure 1:**
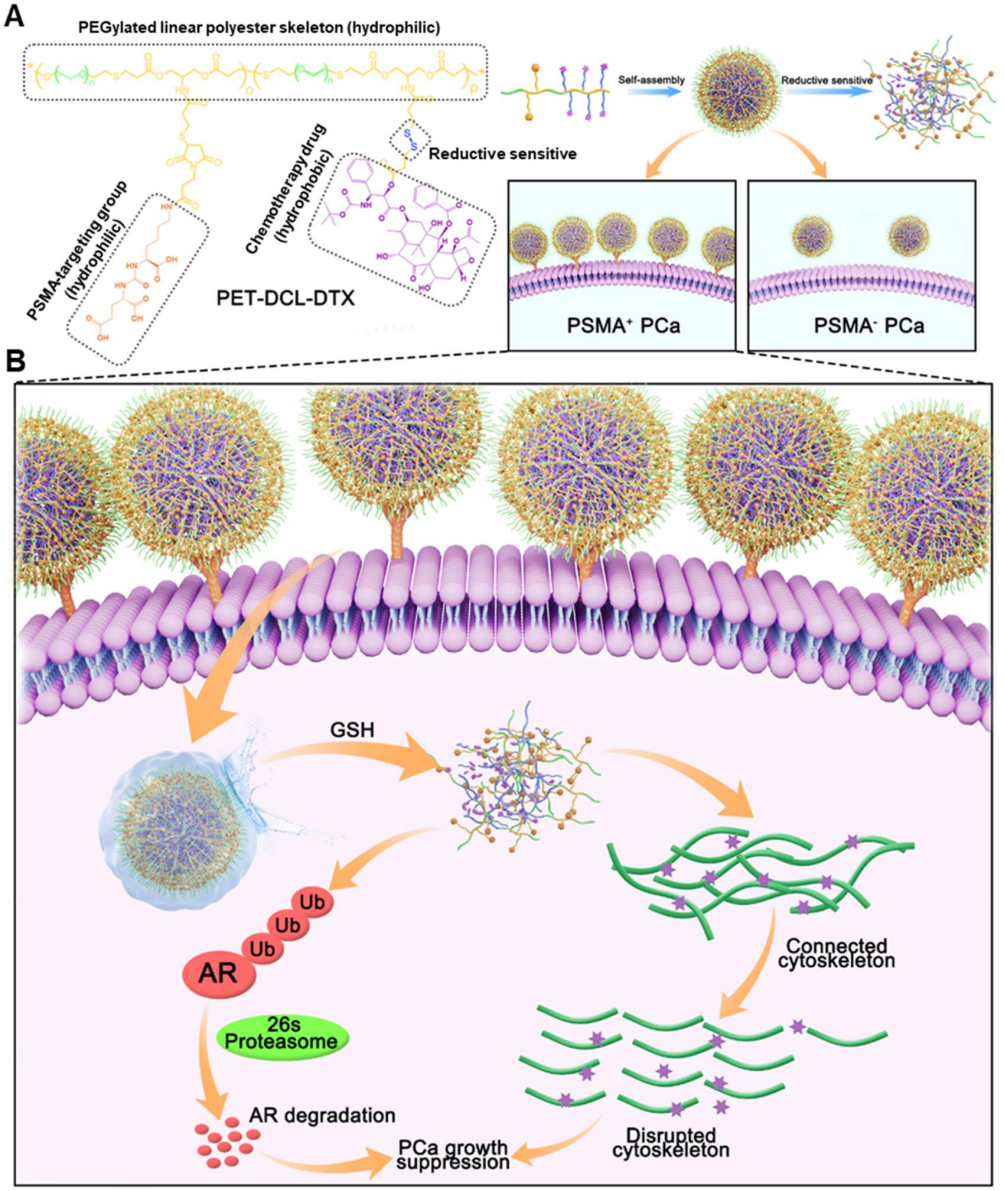
Illustration of PSMA–targeting polyester–DTX nanoconjugate (PET–DCL–DTX). (**A**) Self-assembly into nano-morphology in the water phase owing to the suitable amphipathicity, and degradation and drug release in a reductive environment. (**B**) Specific targeting ability of PSMA-positive PCa cell and thus exerting strong anti-tumor effect.

Therefore, in this study, we plan to use short-chain poly(ethylene glycol) (PEG) as a monomer to directly prepare the polyester rather than as an additional component, aiming to achieve both good water solubility and a robust drug-loading capacity. As is known, PEGylation can improve various properties of the DDSs: (i) the inherent hydrophilicity of PEG can increase the water solubility of DDSs [35]; (ii) the antifouling property endowed by PEG protects DDSs from undesirable uptake *in vivo*, resulting in prolonged circulation and facilitating their optimal utilization of the enhanced permeability and retention (EPR) effects for achieving specific and high accumulation in tumors [36–38]; and (iii) PEGylation can reduce the immunogenicity of the DDSs [39, 40]. In addition, PEGylated DDSs combined with PSMA ligand (2-[3-[5-amino-1-carboxypentyl]-ureido]-pentanedioic acid, DCL) have been successfully used for the targeted therapy of prostate tumors [41, 42]. After careful selection, linear PEG with a molecular weight of 2 kDa was the optimal candidate. The PEG-based polyester was modified with the PSMA targeting ligand DCL, and DTX was covalently introduced via a reduction-sensitive disulfide bond [43, 44], resulting in a novel polymer–drug conjugate (PET–DCL–DTX) (Figure 1A). First, this conjugate can form a near-spherical nano-morphology in water due to its satisfactory amphiphilicity. Hydrophilic PEG and DCL are distributed on the outer layer, while hydrophobic DTX is encapsulated in the core and released in a reductive environment (Figure 1A). Second, with the specific targeting signal, this conjugate could actively recognize PSMA-positive PCa tumor cells (Figure 1B). Third, this conjugate is highly enriched within tumor site via the tumor-inherent EPR effect, while minimizing undesirable distribution to normal tissues. To summarize, our nanoconjugate efficiently enters the PSMA-positive PCa cells and releases DTX via the cleavage of the disulfide bonds in a highly reductive intracellular environment, thereby exerting an anti-cancer activity toward PCa (Figure 1B).

## Materials and methods

### Materials and measurements

All commercial reagents met the experimental requirements and could be used directly without additional purification unless otherwise specified. DTX, 3-maleimidopropionic acid succinimidyl ester (Mal-NHS), N-boc-serinol, 3-(tritylthio) propanoic acid (TrtS-COOH), acryloyl chloride, dicyclohexylcarbodiimide (DCC) and 4-dimethylaminopyridine (DMAP) were purchased from TCI (Japan). 3-(Pyridin-2-yldisulfanyl)propanoic acid (SPDP–COOH), O-(1H-benzotriazol-1-yl)-N,N,N,N-tetramethyluronium hexafluorophosphate (HBTU) and 1-hydroxybenzotriazole (HOBt) were purchased from Shanghai Acmec Biochemical (China). (S)-Di-tert-butyl 2-(3-((S)-6-amino-1-(tert-butoxy)-1-oxohexan-2-yl)ureido)pentanedioate (DCL-O^t^Bu) was purchased from Shanghai Yuanye Bio-Technology (China). Thiol–PEG_2k_–Thiol (SH–PEG–SH) was purchased from Beijing Baikai Biotechnology (China). The characteristics of the polymers including the weight-average molecular weight (MW) and polydispersity index (PDI) were obtained via gel permeation chromatography (GPC, AKTA/FPLC system, GE Healthcare). The ^1^H Nuclear Magnetic Resonance (NMR) and ^13^C NMR spectra were recorded by the NMR instrument (400M, Bruker). The mass spectra (MS) were obtained via electrospray ionization (ESI-TOF MS, Waters Q-TOF Premier) or the matrix assisted laser desorption ionization (MAIDI-HRMS, Autoflex MALDI-TOF/TOF) time-of-flight mass spectrometry. The polymer’s morphology, size and zeta potential were depicted via a transmission electron microscope (TEM, Sartorius Tecnai G2 F20) and the Zetasizer (Malvern, Worcestershire, UK). The DTX content was measured via the ultraviolet (UV) spectrophotometer (METASH, Shanghai). The preparation of the small molecule mid-products and the chemical characterization of the small molecule compounds and polymers except for PET–DCL–DTX are shown in Supplementary Files.

### Preparation of PET–STrt

Under N_2_, the solution of SH–PEG–SH (600 mg, 0.30 mmol), diAC–STrt (158.9 mg, 0.30 mmol) and n-hexamine (6.1 mg, 0.06 mmol) in tetrahydrofuran (THF) (2 mL) was stirred at 25°C for 5 h, and a trace of methyl acrylate was added to quench the polymerization. The reaction solution was then added drop-wise to ether (80 mL) and a solid appeared. After filtration, the solid was washed with ether (20 mL ×3) to yield PET–STrt as a white solid (630 mg, yield: 83.0%) with MW of 19.60 kDa and PDI of 1.37, and ^1^H NMR was used to confirm its chemical structure.

### Synthesis of PET–SH

Under N_2_, CF_3_COOH (2.0 mL) and Et_3_SiH (1.2 mL) were added to a solution of PET–STrt (530 mg) in dichloromethane (38 mL), and the reaction mixture was stirred at ambient temperature for 6 h, then concentrated and washed with ether (20 mL ×3) to obtain PET–SH as a white solid (464 mg) with MW of 17.58 and PDI of 1.32, and ^1^H NMR was used to confirm its chemical structure.

### Preparation of PET–DCL–SH

Under N_2_, Mal-DCL (30 mg) was added to a solution of PET–SH (300 mg) in N,N-dimethylformamide (3 mL), then continuously stirred at ambient temperature for 6 h. The reaction solution was added slowly to reverse osmosis water (RO-water) (30 mL) in an ice-bath used for cooling, followed by dialysis against RO-water (MWCO = 8 kDa), filtration and lyophilization to yield PET–DCL–SH as a white solid (270 mg) with MW of 19.84 and PDI of 1.43, and ^1^H NMR was used to confirm the chemical structure.

### Synthesis of PET–DCL–DTX

Under N_2_, SPDP–DTX (40 mg) was added to a solution of PET–DCL–SH (100 mg) in N,N-dimethylformamide (2 mL), then continuously stirred at ambient temperature for 12 h. Methyl acrylate (20 mg) was added to block the unreacted thiols. The reaction solution was added slowly to RO-water (20 mL) in an ice-bath used for cooling, followed by dialysis against RO-water (MWCO = 8 kDa), filtration and lyophilization to yield PET–DCL–DTX as a white solid (78 mg) with MW of 23.36 and PDI of 1.38, and ^1^H NMR was used to confirm its chemical structure.

### Determination of DTX content

The DTX content was determined by a UV spectrophotometer (METASH, Shanghai) at an absorption wavelength of 230 nm. A standard curve created by measuring the absorption values of different concentrations of DTX (0.39–6.25 mg/mL) was utilized to determine the DTX content in PET–DCL–DTX, which was diluted using a H_2_O:CH_3_OH (v/v = 1:1) solvent.

### *In vitro* release of DTX

To evaluate the reduction-sensitive DTX release from PET–DCL–DTX, two different physiological reduction conditions were simulated *in vitro*, including tumor cells (10 mM of glutathione (GSH) in PBS, pH 5.4, 37°C) and normal cells (10 μM of GSH in PBS, pH 7.4, 37°C). PET–DCL–DTX (4.30 mg, with 300 μg of DTX) was dissolved in the above two dithiothreitol (DTT) solutions, then they were incubated at 37°C for 48 h. During this process, 200 μL of the sample were obtained at different time points (0 h, 1 h, 2 h, 4 h, 8 h, 12 h, 24 h, 48 h). The samples taken out were diluted with 400 μL of methanol and divided into triplicates; the DTX concentrations were then determined via HPLC, which was used for evaluating the DTX release behavior of PET–DCL–DTX. Additionally, the same method was used to study the effects of different acidic environments (PBS, pH 7.4, 37°C or PBS, pH 5.4, 37°C) on drug release. The conditions of HPLC: the column is Shim-pack GLST C18 (Shimadzu, Japan) packed with 5 μm particle size and its column size is 250 × 4.6 mm. The mobile phase is CH_3_OH:H_2_O (*v*/*v* = 7:3). The flow rate, the column temperature and the injection volume are set to 1.0 mL/min, 35°C and 20 μL, respectively.

### Cell culture

PCa cell lines including C4-2, CWR22Rv1 and PC3 were purchased from Xiamen Immocell Biotechnology Co., Ltd. They were cultured in 10% certified FBS RPMI medium (VivaCell, Shanghai, China) supplemented with 2 mM L-glutamine, 100 IU/mL penicillin and 100 μg/mL streptomycin.

### *In vivo* animal model

This animal experiment was performed under the approval from the animal committee of southwest medical university. Six- to eight-week-old nude mice were purchased from the Chongqing Tengxin Laboratory Animal Co., Ltd (Chongqing, China) and subcutaneously injected with 1 × 10^6^ CWR22Rv1 cells (1:1 with growth factor reduced matrigel). When the tumors reached an average size, the mice were grouped and administrated with (i) PET–DCL–DTX, (ii) PET–DCL–SH, and (iii) DTX. The effective concentration of DTX was 5 mg/kg. DTX was dissolved in 0.9% saline and PET–DCL–DTX was dissolved in corn oil. Both drugs were intraperitoneally injected into mice every other day for 1 month. The body weight, tumor size and tumor weight were measured as described in the Results section. The tumor volume was calculated using the formula *V* = ab^2^/2 (cm^3^). When the mice were sacrificed, each tumor was removed and stored in liquid nitrogen for further use. This animal experiment was performed under the approval from the animal committee of Southwest Medical University (swmu20250571). Other methods are listed in the Supplementary Files.

### Statistical analysis

The data were expressed as mean ± SEM (the standard error of the mean) from at least three independent experiments. The statistical analyses, which involved one-way ANOVA followed by a *t*-test, were performed in SPSS 17.0 software. *P* < 0.05 was considered as statistically significant.

## Results and discussion

### Preparation and characterization

**Figure 2:**
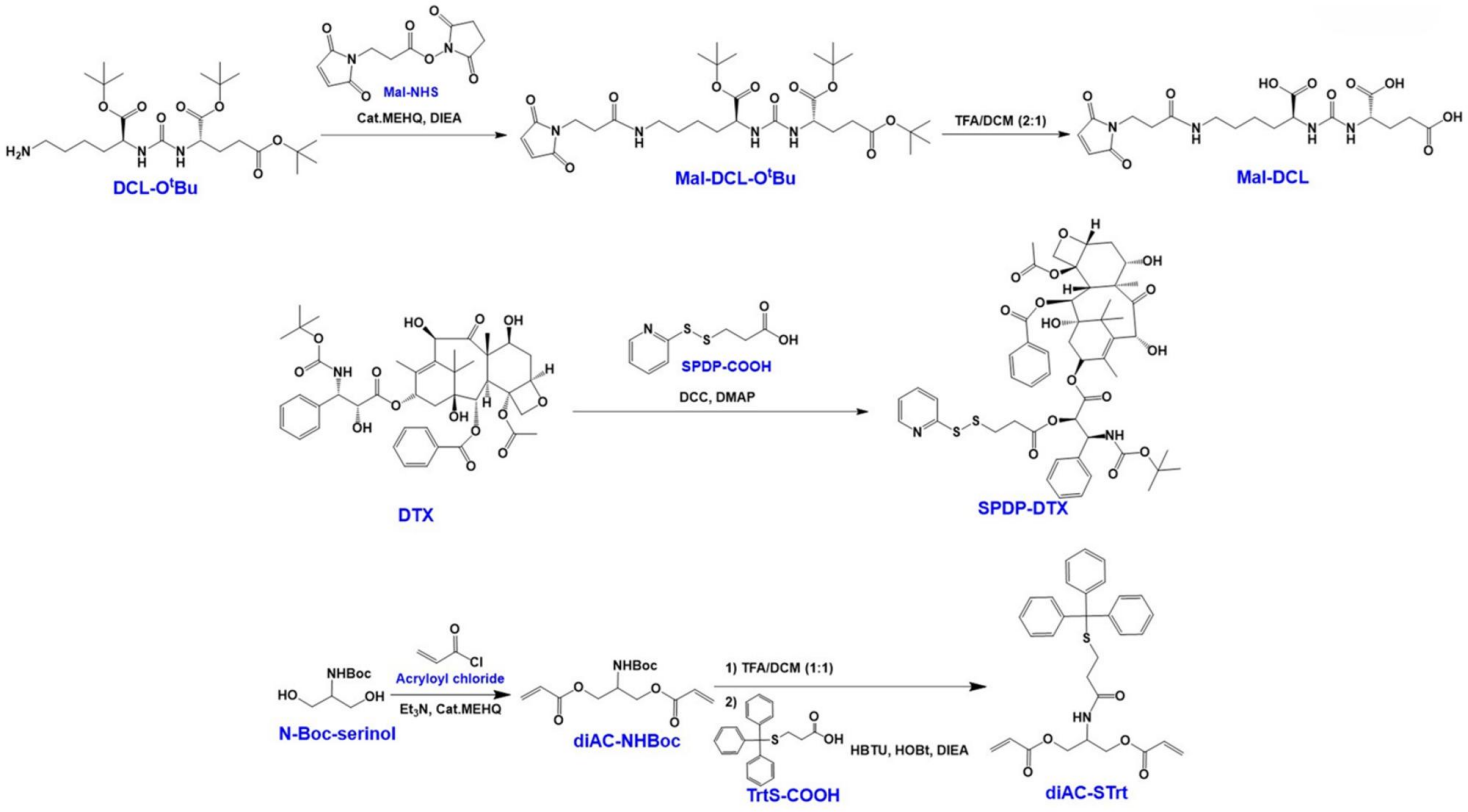
Synthesis process of Mal-DCL, SPDP–DTX, and diAC–STrt.

**Figure 3:**
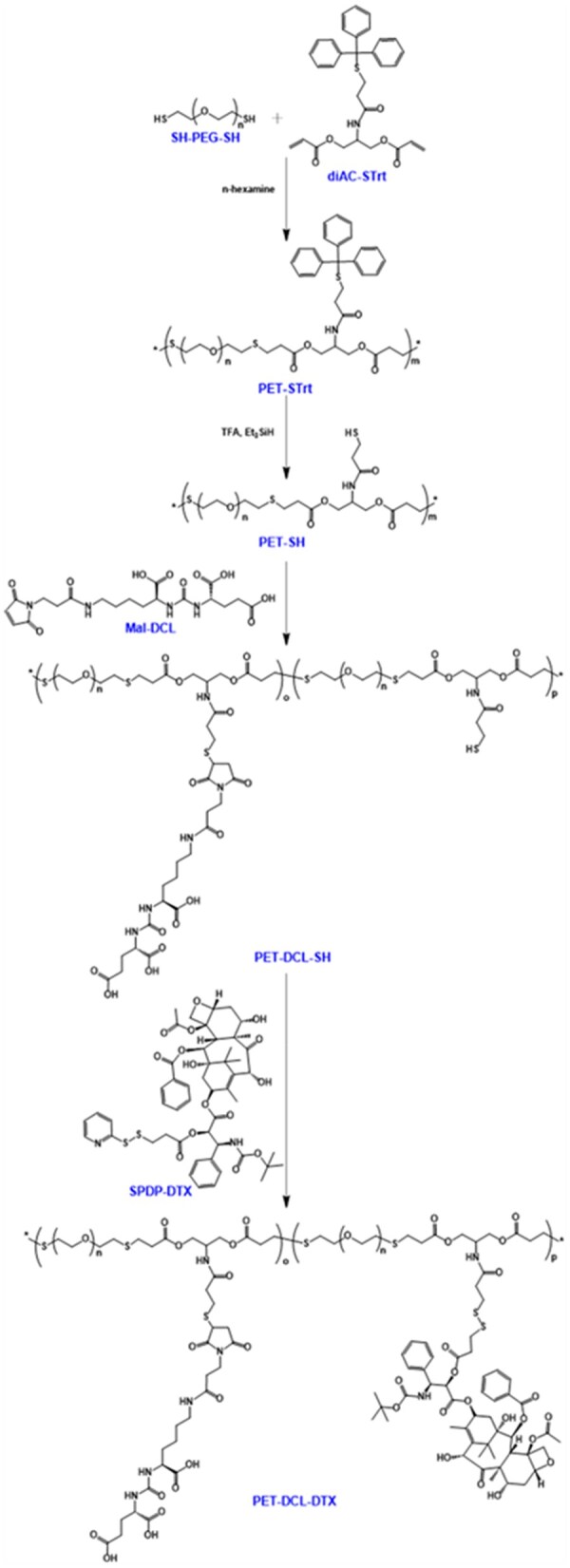
Synthesis process of PET–DCL–DTX.

**Figure 4:**
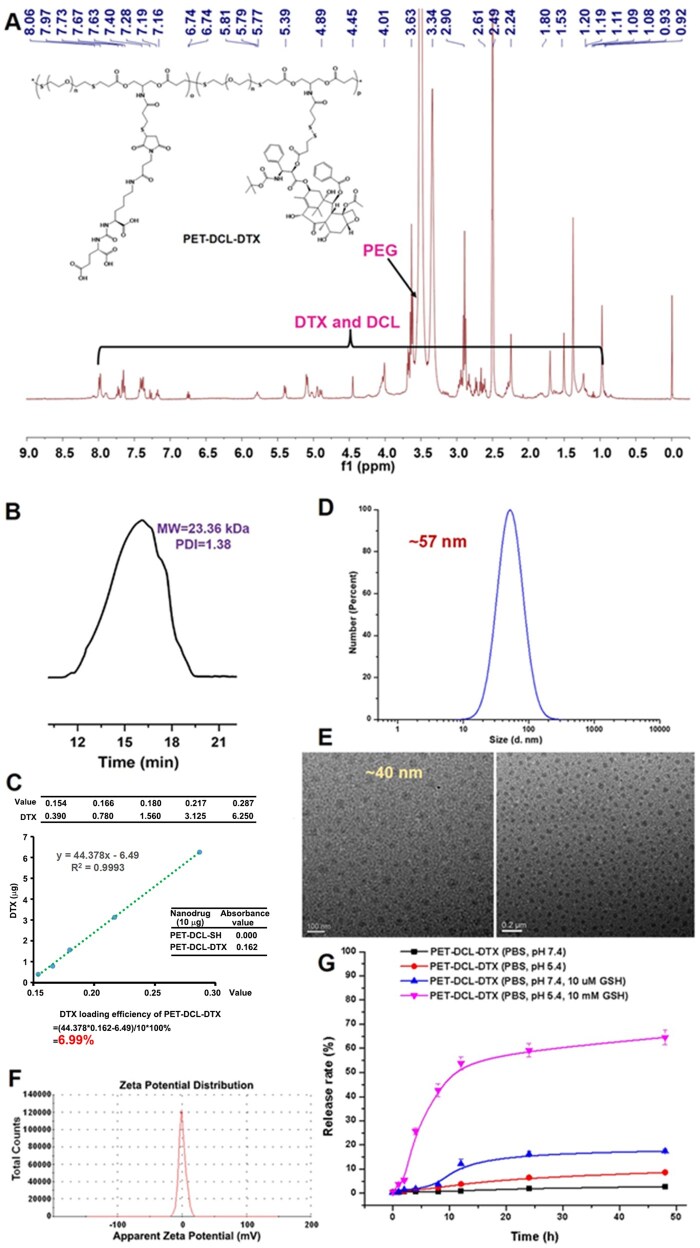
Chemical characterizations of PET–DCL–DTX. (**A**) ^1^H NMR spectrum (recorded in *d*_6_-DMSO). (**B**) Molecular weight and polymer dispersity index (MW = 23.36 kDa and PDI = 1.38, measured via GPC). (**C**) DTX content (6.99%, confirmed via UV). (**D**) Particle size in aqueous phase (∼57 nm, detected by DLS). (**E**) Morphology (near-spheroid, ∼40 nm, detected via TEM). (**F**) Surface potential (∼0 mV, measured via DLS). (**G**) DTX release profiles of PET–DCL–DTX under different conditions: PBS (pH 7.4, at 37°C), PBS (pH 5.4, at 37°C), PBS with10 μM GSH (pH 7.4, at 37°C) or PBS with 10 mM GSH (pH 5.4, at 37°C).

In light of the challenges identified in our previous study, we synthesized a water-soluble polyester using linear PEG with enhanced hydrophilicity as a monomer. Subsequently, the PSMA targeting group DCL and hydrophobic DTX were simultaneously covalently introduced to prepare a novel polyester-based DDS (PET–DCL–DTX) for the treatment of PSMA^+^ PCa (Figures 2 and 3). Since both the DCL and DTX need to be conjugated to the polyester through covalent bonds, their functional modifications were necessary. Referring to our previous study [12], maleimide-modified DCL prodrug (Mal-DCL) and SPDP-modified DTX prodrug (SPDP–DTX) were prepared, they can be covalently conjugated to the carrier through the thiol-ene click reaction and the reduction-sensitive disulfide bond, respectively. In the preparation of the polyester carrier, a functional monomer containing diacrylate and protected thiol (diAC–STrt) was synthesized in a two-step reaction. First, N-Boc-serinol was di-esterified by acryloyl chloride to obtain diAC–NHBoc and it was confirmed by NMR, MS analysis and HPLC (Supplementary Figures S1–S5). Next, the Boc protecting group of diAC–NHBoc was removed by CF_3_COOH and then condensed with TrtS–COOH to obtain diAC–STrt. By comparing the ^1^H NMR spectra before and after the reaction (Supplementary Figures S1 and S6), we observed that the disappearance of the tert-butyl group was accompanied by the appearance of Trt. Additionally, ^13^C NMR, MS analysis and HPLC (Supplementary Figures S7–S10) further confirmed its chemical structure. To overcome the poor water solubility of polyester as the carrier, this study utilized short-chain PEG as the hydrophilic monomer for polymerization. The selection principle for the PEG chain length is to enhance water solubility while ensuring an appropriate degree of polymerization and a suitable proportion of functional monomers. Following the initial screening, PEG with MW of 2 kDa was finally selected. Linear PEG with thiols at both ends (SH–PEG–SH) and the diacrylate monomer (diAC–STrt) underwent thiol-ene click polymerization catalyzed by n-hexamine at 25°C. Here, a monomer ratio of 1:1 was utilized to facilitate both high polymerization and low PDI, as established by previous studies [34, 45]. The crude product was then purified via ether washing to obtain the pure target copolymer (PET–STrt), because both monomers are easily soluble in ether and the copolymer is not. In the ^1^H NMR spectrum (Supplementary Figure S11), all the peaks appear broad, which is a characteristic of the polymer, and the hydrogen signals of PEG and Trt appeared in pairs, whereas the alkene hydrogen signals were not found at 6.0–6.5 ppm. Additionally, GPC analysis showed that its MW and PDI were 19.60 kDa and 1.37 (Supplementary Figure S12A), respectively. Here, a larger PDI was also a typical characteristic of this thiol-ene step-growth polymerization [46]. Additionally, dynamic light scattering (DLS) analysis (Supplementary Figure S12B) showed that PET–STrt has good amphiphilicity and can be assembled into stable nanostructures (∼32 nm) in aqueous phase. The above results indicated the success of the polymerization. Next, PET–STrt was treated with CF_3_COOH and Et_3_SiH to remove Trt and the thiols-exposing water-soluble copolymer (PET–SH) was obtained. The ^1^H NMR result clearly showed that the Trt hydrogen signals were missing (Supplementary Figure S13) and the MW was correspondingly reduced to 17.58 kDa (Supplementary Figure S14A). In addition, after the detachment of hydrophobic Trt, PET–SH becomes too hydrophilic and cannot form compact and stable nanoparticles in the aqueous phase (Supplementary Figure S14B). Subsequently, DCL and DTX were covalently introduced successively with PET–SH as the carrier. First, Mal-DCL was bound to PET–SH via thiol-ene click chemistry for the preparation of PET–DCL–SH. Here, the amount of Mal-DCL was controlled at 10 wt% of the carrier, which could not only meet the targeting requirements, but also retain more thiols to achieve the subsequent high drug loading. The chemical structure of PET–DCL–SH was characterized by the ^1^H NMR and GPC analysis. The characteristic peaks of DCL (carboxyl, amide and carbonylamide) were observed in the ^1^H NMR spectrum and the characteristic peaks of PEG were retained (Supplementary Figure S15); this indicated successful introduction of DCL. Moreover, the increase of MW in the GPC analysis (Supplementary Figure S16A) also supported this result. Since DCL is also hydrophilic, PET–DCL–SH, like PET–SH, lacks good hydrophilic and hydrophobic balance, and its aqueous phase particle size is also large and unstable (Supplementary Figure S16B). After the introduction of DCL, DTX was introduced onto the carrier via thiol-disulfide exchange chemistry between SPDP–DTX and PET–DCL–SH, finally preparing the target polyester–DTX conjugate (PET–DCL–DTX). In this step, both the maximum drug loading and water solubility were considered, hence the dosage ratio of SPDP–DTX and PET–DCL–SH should be titrated. The input amount of hydrophobic SPDP–DTX was finally determined to be 40 wt% of PET–DCL–SH. The introduction of DTX was clearly demonstrated in the ^1^H NMR analysis (Figure 4A), and as a result, its MW also increased from 19.84 to 23.36 kDa (Figure 4B). Crucially, PET–DCL–DTX had a considerable drug loading of 6.99% (Figure 4C) while maintaining water solubility greater than 10 mg/mL. Additionally, this conjugate was amphiphilic and could self-assemble to form nanostructures in water systems. The DLS particle size (ca. 57 nm) (Figure 4D) was larger than the TEM particle size (ca. 40 nm) (Figure 4E). This difference was attributed to the DLS measurements being conducted in an aqueous solution, where the polymer was in the expanded state, while the TEM analysis was performed in the dry state, resulting in the polymer being contracted. Furthermore, its surface potential was approximately 0 mV (Figure 4F). Of note, the antifouling property endowed by PEG can help the nanoconjugate escape the undesired uptake *in vivo* [37, 38]. Additionally, DCL is hydrophilic, so after self-assembly, it is theoretically exposed on the outside thereby exerting its targeting ability toward PSMA. For supporting this, we conducted X-ray photoelectron spectroscopy (XPS) analysis on PET–DCL–DTX. First, a wide C1s peak appeared between 288.0 and 290.0 eV (Supplementary Figure S17A), indicating the presence of multiple carbonyl-containing components on its surface, including carboxyl, carbonylamide and ester groups. Second, in the N1s curve, the characteristic peak of carbonylamide was observed at approximately 401.0 eV (Supplementary Figure S17B). These results confirm that DCL is exposed on the surface after self-assembly of PTE–DCL–DTX.

### *In vitro* release of DTX

In this study, DTX was conjugated onto the polymer via reduction-sensitive disulfide bonds to enable stimulus-sensitive release, leveraging the higher reducibility of tumor cells compared to normal cells. Herein, two different physiological reduction conditions (tumor cells: 10 mM of GSH in PBS, pH 5.4, 37°C or normal cells: 10 µM of GSH in PBS, pH 7.4, 37°C) were simulated *in vitro*. PET–DCL–DTX was then incubated in these conditions, and the change of the DTX concentration was monitored during a 48-h incubation period by high performance liquid chromatography (HPLC) to evaluate the release behavior of DTX. The results (Figure 4G) revealed that DTX was rapidly released from the polymer under high GSH concentration, reaching ∼53% in the first 12 h and ∼65% after 48 h. In contrast, DTX release was significantly slower under low GSH concentration, with only ∼12% and ∼17% release at 12 and 48 h, respectively. The above results indicate that PET–DCL–DTX maintains high stability under normal physiological reduction condition while releasing drugs rapidly and efficiently under high tumor reduction condition.

Additionally, the impact of different pH values (PBS, pH 7.4, 37°C or PBS, pH 5.4, 37°C) on the DTX release was also evaluated. The results (Figure 4G) showed that PET–DCL–DTX remained relatively stable under normal physiological pH condition, with a total release amount of less than 3%. However, under acidic condition, the release efficiency of DTX was improved, with a total release amount of ∼9% owing to the hydrolysis of the ester bonds. These results indicate that slight acidity can promote DTX release from the polymer through the hydrolysis of ester bonds.

### Polyester–DTX nanoconjugate specifically suppresses PSMA-positive PCa cell growth

**Figure 5:**
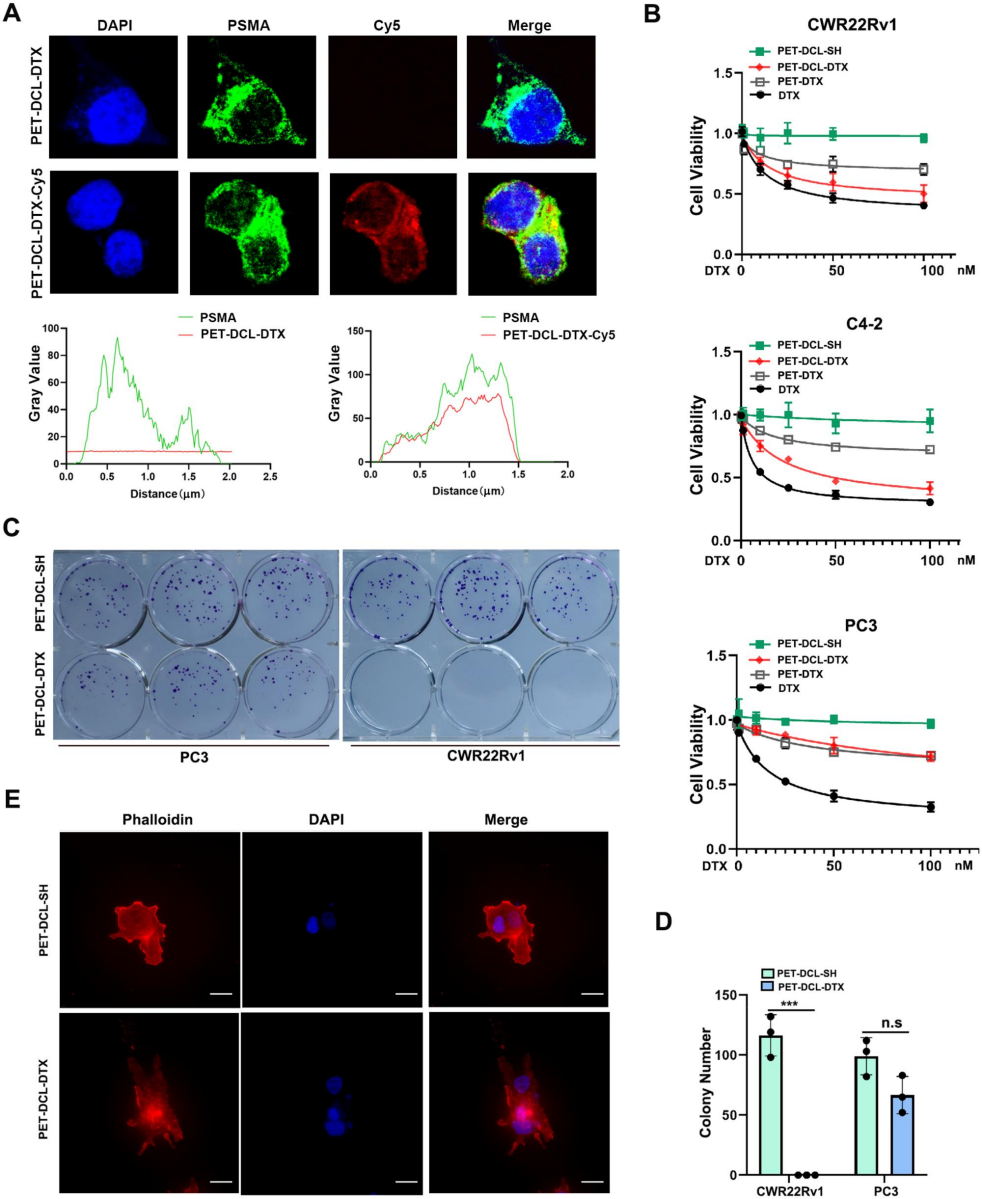
Polyester–DTX nanoconjugate specifically suppresses PSMA-positive PCa cell growth. (**A**) Confocal microscopy of PSMA^+^ CWR22Rv1 cells treated with PET–DCL–DTX or Cy5 labeled PET–DCL–DTX for 4 h. (**B**) Cell viability of CWR22Rv1, C4-2 and PC3 cells treated with various concentrations of PET–DCL–DTX, PET–DCL–SH, PET–DTX and DTX. (**C**) Colony formation assay of CWR22Rv1 and PC3 cells treated with 10 nM PET–DCL–DTX or the drug-free carrier. (**D**) Statistical analyses of **C**. (**E**) F-actin staining of CWR22Rv1 cells treated with 10 nM PET–DCL–DTX for 24 h. Scale bar: 5 μm. *** p < 0.001.

We first examined the PSMA specific recognition of the constructed polyester–DTX nanoconjugate PET–DCL–DTX. We performed the confocal microscopy to examine the co-localization of Cy5 labeled PET–DCL–DTX with PSMA in CWR22Rv1 cells, showing that PET–DCL–DTX has a strong localization signal with PSMA (Figure 5A), highly suggesting that PET–DCL–DTX specifically recognizes PSMA to exert its biological functions. Next, the cancer suppressive ability of PET–DCL–DTX was closely evaluated: PSMA-positive PCa cells including CWR22Rv1 and C4-2 were treated with different concentration of PET–DCL–DTX and its carrier control PET–DCL–SH. In addition, the anti-cancer efficacy of PET–DCL–DTX was compared to that of the naked DTX drug and its specific targeting ability was confirmed in PSMA negative PC3 cells. As shown in Figure 5B, PET–DCL–DTX remarkably suppressed the cell growth of CWR22Rv1 and C4-2 in a dose-dependent manner as compared to PET–DCL–SH, with IC_50_ values of 21.82 and 24.55 nM, respectively. In contrast, the suppressive activity of PET–DCL–DTX on the PSMA negative PC3 cells was minimal, with IC_50_ of 138.10 nM, whereas DTX dramatically inhibited PCa cell viability without PSMA selection (Figure 5B), with 14.39, 6.08 and 18.00 nM in CWR22Rv1, C4-2 and PC3, respectively. To compare the passive and active targeting effects, we also constructed polymer–DTX conjugate without DCL (PET–DTX) and used it to treat various PCa cells. As shown in Figure 5B, PET–DTX, with an 8.24% DTX loading capacity (data not shown), had similar anti-cancer activity toward C4-2, CWR22Rv1 and PC3 cells, suggesting it passively enters cells without PSMA selectivity to exert the anti-cancer activity. Moreover, cell viability inhibition by PET–DTX is much less than that by PET–DCL–DTX in PSMA-positive C4-2 and CWR22Rv1 cells (Figure 5B), indicating PSMA-positive cells actively respond to PET–DCL–DTX. The anti-cancer activity of PET–DCL–DTX on PCa cells was also confirmed by a colony formation assay, which also demonstrated that PET–DCL–DTX remarkably suppressed the colony formation ability of CWR22Rv1 cells but had a negligible effect on the PC3 cells (Figure 5C and D) compared to the drug-free carrier. Interestingly, we found that the GSH/GSSG ratio in PSMA-positive PCa cells (C4-2, C4-2B and CWR22Rv1 cells) is much higher than that in PSMA negative PC3 and Du145 cells (Supplementary Figure S18), implying this nanodrug may have a faster sensitive release in PSMA-positive PCa cells. However, whether this reductive environment is regulated by PSMA or AR deserves future exploration. These data collectively suggest that PET–DCL–DTX specifically recognizes the PSMA antigen to exert its anti-cancer activity.

Because DTX is a clinically well-known drug that disrupts the cellular cytoskeleton structure by binding β-tubulin, we sought to determine whether the DTX packaged PET–DCL–DTX still harbored this ability. As expected, this nanoconjugate strongly disrupted the multi-branched microtubular networks of the CWR22Rv1 cells compared to the drug-free vehicle (Figure 5E), suggesting that a disrupted cytoskeleton network is one cause of PET–DCL–DTX induced cell death. Collectively, all these results suggest that the anti-cancer activity of PET–DCL–DTX on the PCa cell growth relies on its PSMA recognition.

### Polyester–DTX nanoconjugate constrains PCa cell growth by regulating cell proliferation and cell apoptosis

**Figure 6:**
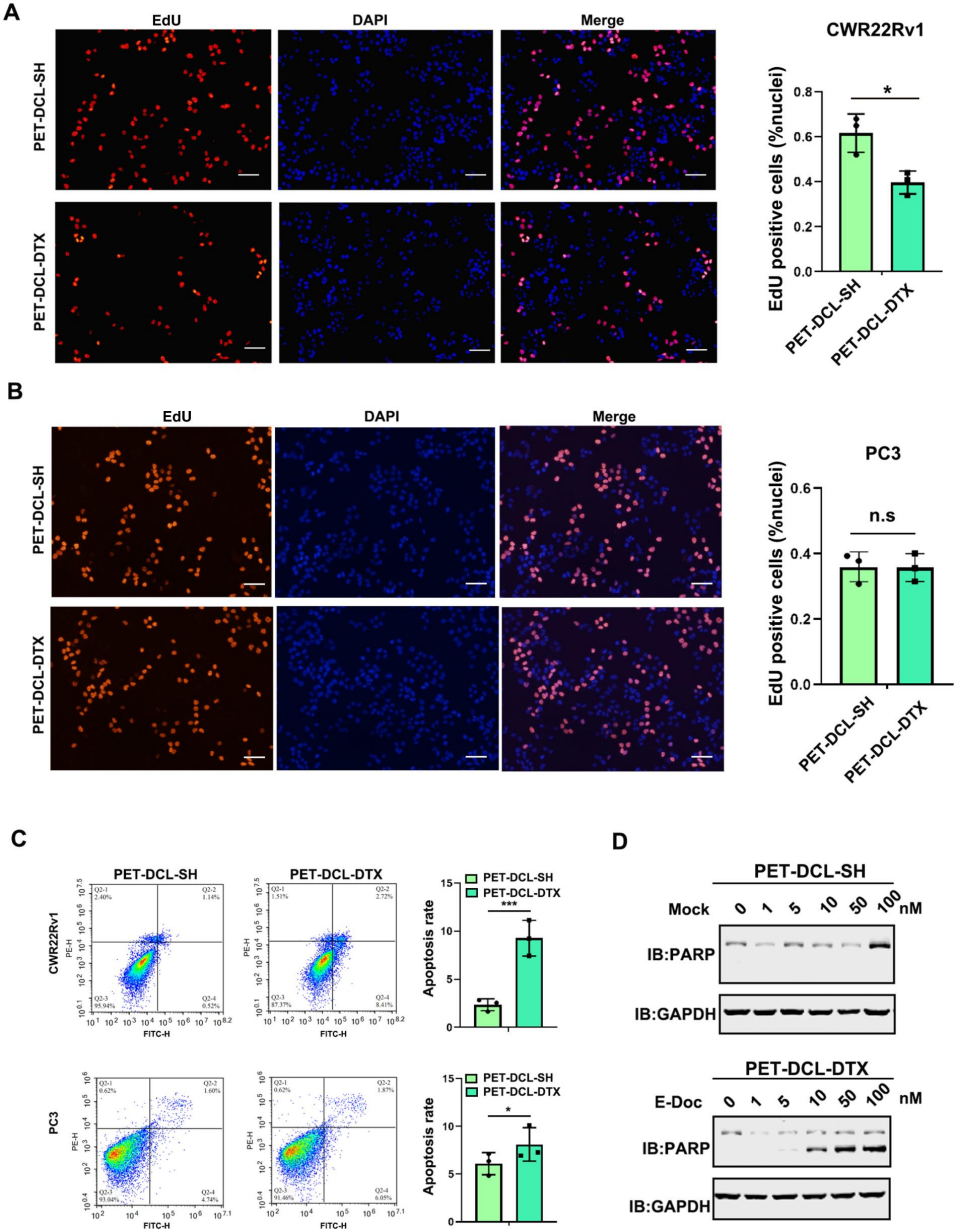
Polyester–DTX nanoconjugate constrains PCa cell growth by regulating cell proliferation and cell apoptosis. EdU staining of (**A**) CWR22Rv1 and (**B**) PC3 cells treated with 10 nM PET–DCL–DTX or the drug-free carrier for 24 h. DAPI was used to monitor the nuclei. Scale bar: 100 μm. Left, representative images of EdU staining. Right, statistical analysis of EdU staining. (**C**) Apoptosis of CWR22Rv1 (top) and PC3 cells (bottom) with and without 10 nM PET–DCL–DTX treatment for 24 h. Left, representative images from flow cytometry. Right, statistical analyses. (**D**) Western blotting detection of cleaved PARP in CWR22Rv1 cells treated with 10 nM PET–DCL–DTX and drug-free carrier for 24 h. * p < 0.05.

Apoptosis and proliferation are two critical biological events in cells. We first examined whether PET–DCL–DTX treatment could affect the cell proliferation of PCa cells, monitored by EdU staining assay. The results showed that the number of EdU positive cells was substantially decreased upon PET–DCL–DTX exposure than that of the cells treated with drug-free PET–DCL–SH (Figure 6A, 61.6% versus 39.6%). In contrast, the percentage of EdU positive cells was comparable in PSMA-nul PC3 cells with or without PET–DCL–DTX treatment (Figure 6B), suggesting that this nanodrug specially recognizes the PSMA antigen and exerts its cell proliferation inhibition. Subsequently, we sought to determine whether PET–DCL–DTX could trigger the cell apoptosis of PCa cells. To this end, PSMA-positive CWR22Rv1 cells and PSMA negative PC3 were exposed to 10 nM PET–DCL–DTX treatment for 48 h and collected for the annexin V/Flow cytometric assay. As shown in Figure 6C, PET–DCL–DTX had a stronger capacity to increase the CWR22Rv1 cell apoptosis than PET–DCL–SH (9.2% versus 2.3%), which was also confirmed by the western blotting detection of the apoptotic biomarker, cleaved PARP (Figure 6D). However, the apoptosis triggering activity of PET–DCL–DTX was not obviously observed in the PSMA negative PC3 cells (6.0% versus 8.1%, Figure 6D). These data illustrated that PET–DCL–DTX specifically suppresses the cell viability of PSMA-positive PCa cells with its targeting DCL moiety and loaded DTX by triggering cell apoptosis, blocking cell cycle entry and slowing down the cell proliferation rate.

### PET–DCL–DTX inhibits cell migration and cell invasion of PSMA PCa cells

**Figure 7:**
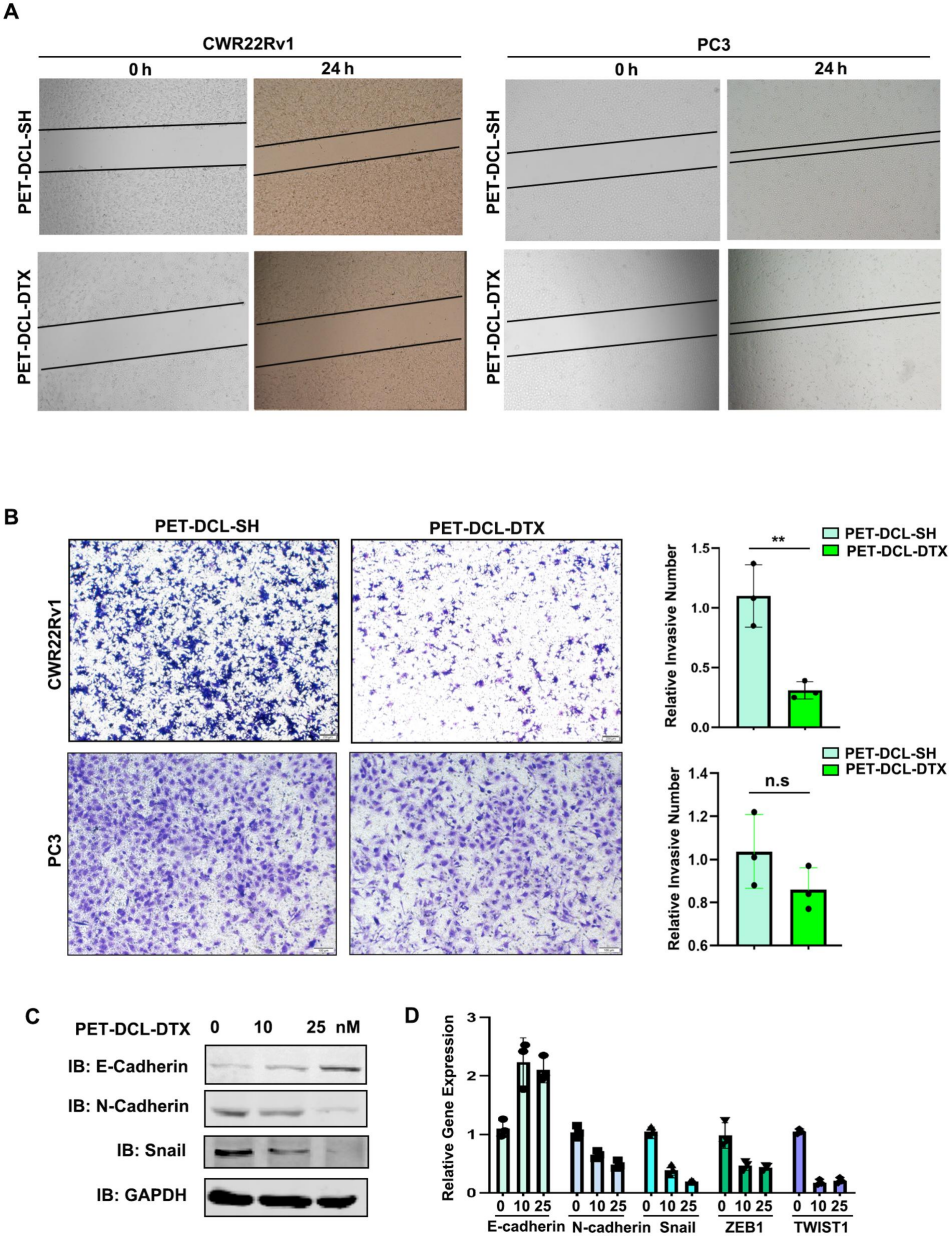
PET–DCL–DTX inhibits cell migration and cell invasion of PSMA PCa cells. (**A**) Wound healing assay of CWR22Rv1 (left) and PC3 cells (right) treated with 10 nM PET–DCL–DTX and the drug-free carrier for 24 h. (**B**) Transwell invasion assay of CWR22Rv1 (top) and PC3 cells (bottom) treated with 10 nM PET–DCL–DTX and the drug-free carrier for 24 h. Left, representative images of invading cells. Right, a statistical analysis. Scale bar: 200 μm. (**C**) Western blotting to detect E-cadherin, N-cadherin and Snail. GAPDH was the loading control. (**D**) Real-time PCR to detect mRNA levels of EMT related genes before and after 10 nM PET–DCL–DTX treatment. The expression level of the interested genes was normalized to GAPDH mRNA. ** p < 0.01.

Next, we examined whether PET–DCL–DTX influenced the cell migration and cell invasion of PSMA PCa cells. As exhibited in Figure 7A, PET–DCL–DTX powerfully suppressed the cell migration of the CWR22Rv1 cells, monitored by a wound healing assay. However, this inhibition on the cell migration was not observed in PSMA null PC3 cells (Figure 7A). Moreover, the transwell invasion assay revealed that PET–DCL–DTX treatment led to a strong cell invasion inhibition of the CWR22Rv1 cells (Figure 7B, top). As expected, PET–DCL–DTX lost its impact on the cell invasion of PC3 cells (Figure 7B, bottom). Epithelial–mesenchymal transition (EMT) is a key step during cancer migration and invasion. We noted that PET–DCL–DTX suppressed the EMT process of PSMA PCa cells (Figure 7C), which showed that the expression level of E-cadherin (epithelial marker) was increased while the expression level of N-cadherin (mesenchymal marker) and Snail were decreased after PET–DCL–DTX treatment. Moreover, PET–DCL–DTX treatment led to EMT blockage, as demonstrated by the changes in the mRNA levels of EMT related genes (Figure 7D). Collectively, these results suggest that PET–DCL–DTX harbors a strong inhibition on the cell migration and cell invasion of PSMA-positive PCa cells owing to its DCL moiety and packaged DTX.

### PET–DCL–DTX mediates AR reduction via ubiquitin-proteasome degradation pathway

**Figure 8:**
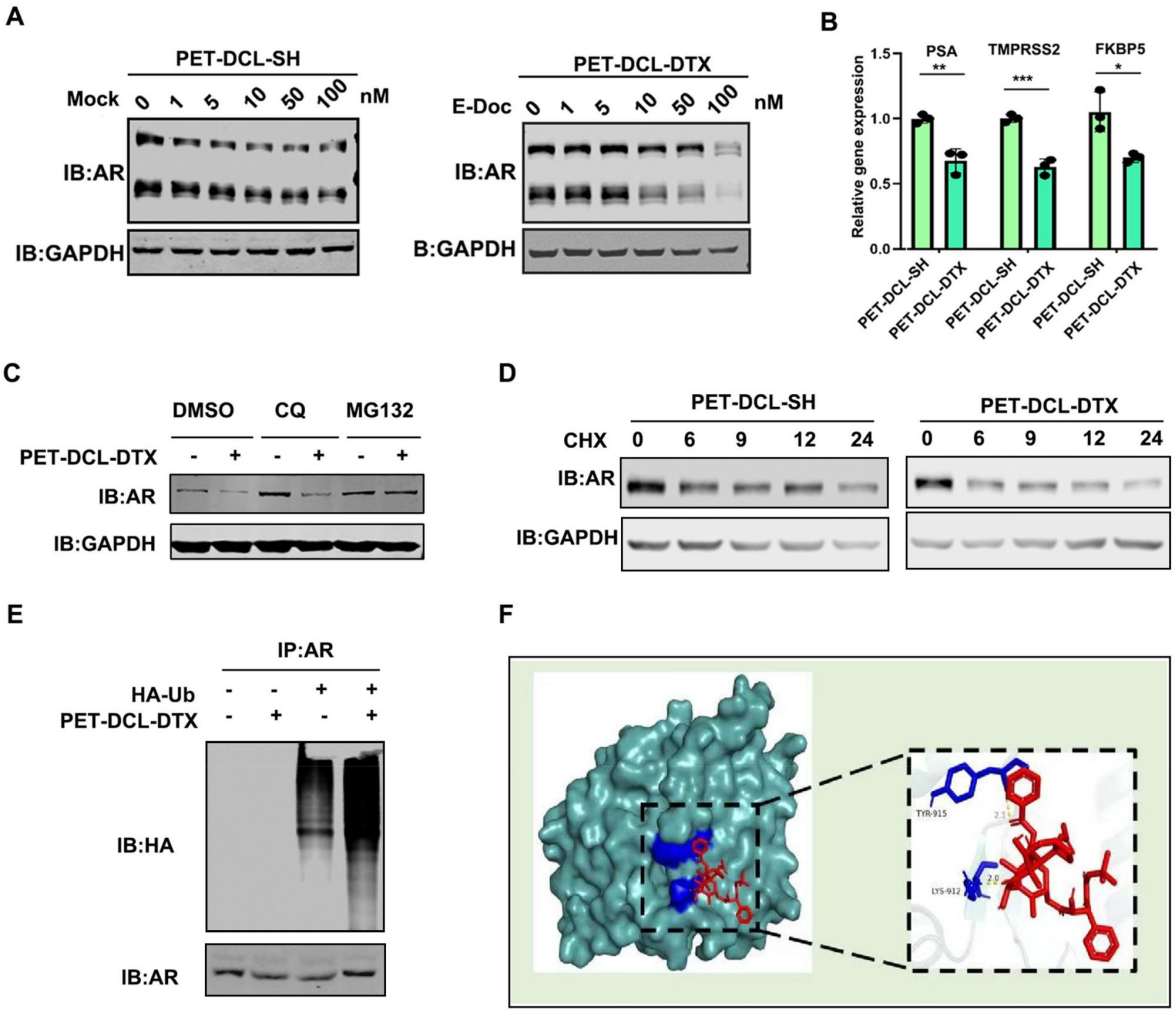
PET–DCL–DTX mediates AR reduction via ubiquitin-proteasome degradation pathway. (**A**) AR protein level examined by Western blotting in CWR22Rv1 cells treated with 10 nM PET–DCL–DTX or the drug-free carrier for 24 h. GAPDH was used as the loading control. (**B**) The expression levels of AR targeted genes monitored by qPCR in CWR22Rv1 cells treated with PET–DCL–DTX or the drug-free carrier for 24 h. Gene expression was normalized to GAPDH mRNA. (**C**) MG132 treatment blocked PET–DCL–DTX induced AR degradation. GAPDH was the internal control. (**D**) AR degradation rate with or without PET–DCL–DTX treatment. GAPDH was utilized as the loading control. (**E**) Ubiquitination assay of AR with or without PET–DCL–DTX treatment. (**F**) DTX may interact with AR, as predicted by the AutoDock program. * p < 0.05,** p < 0.01, *** p < 0.001.

Given the facts that AR signaling is the main driving force determining PCa development and previous reports have shown that AR is also a target of DTX, we proposed to determine whether PET–DCL–DTX still harbors the capacity to reduce AR expression. As shown in Figure 8A, 48-h exposure of PET–DCL–DTX remarkably led to AR reduction in the CWR22Rv1 cells in a dose-dependent manner. In contrast, the drug-free carrier had no impact on the AR protein level. AR signaling is presented by the transcriptional regulation of its downstream genes. In line with AR reduction, the mRNA levels of AR downstream targets including PSA, TMPRSS2 and FKBP5 were also significantly suppressed by PET–DCL–DTX but not by PET–DCL–SH treatment (Figure 8B). We also observed that AR reduction by PET–DCL–DTX occurred in the proteasome because its inhibitor MG132, but not the lysosome inhibitor chloroquine, prevented AR degradation (Figure 8C). In addition, PET–DCL–DTX treatment significantly enhanced the AR degradation rate (Figure 8D). Moreover, more ubiquitin was conjugated to AR when CWR22Rv1 cells were exposed to PET–DCL–DTX treatment (Figure 8E), suggesting that AR reduction by this nanodrug is mediated by the ubiquitin-proteasome degradation pathway. Possibly, DTX released from the PET–DCL–DTX nanodrug may directly interact with AR (Figure 8F) as predicted by Autodock and alter its conformation. We conclude that PET–DCL–DTX inhibits AR signaling to constrain PSMA PCa progression.

### Polyester–DTX nanoconjugate retards PSMA-positive PCa tumor growth *in vivo*

**Figure 9:**
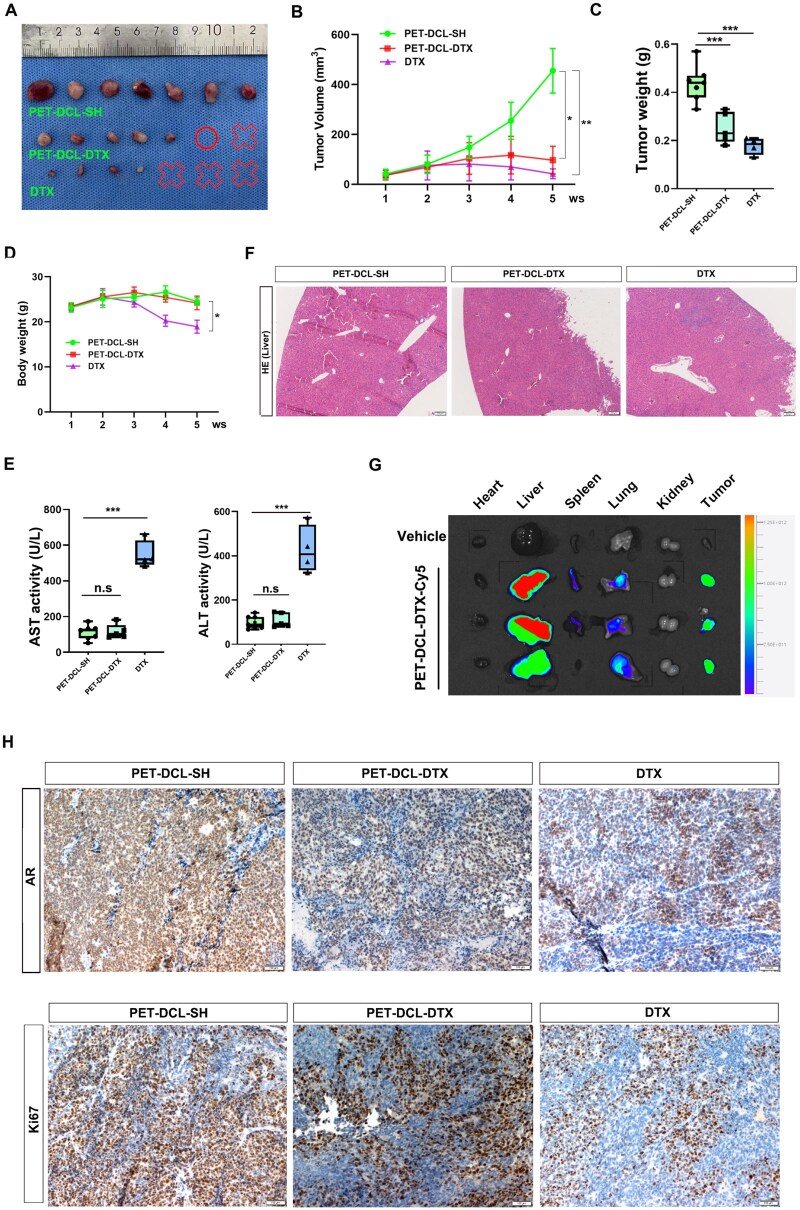
Polyester–DTX nanoconjugate retards PSMA-positive PCa tumor growth *in vivo*. (**A**) Tumor images at the endpoint of therapies. Circle symbol: disappeared PCa tumors. Error symbol: PCa tumors in dead mice. (**B**) Growth curves of CWR22Rv1 tumors, which received PET–DCL–DTX, PET–DCL–SH or DTX administration. (**C**) Tumor weight at the endpoints of the therapies. (**D**) Body weight curves of CWR22Rv1 bearing mice which received PET–DCL–DTX, PET–DCL–SH or DTX therapies. (**E**) ALT and AST levels in serum plasma from PET–DCL–DTX, PET–DCL–SH or DTX treated mice. (**F**) Histological HE staining of livers from PET–DCL–DTX, PET–DCL–SH or DTX treated mice. Scale bar: 200 μm. (**G**) IVIS system to measure the distribution of PET–DCL–DTX in xenograft PCa model. (**H**) IHC staining of AR and Ki67 in CWR22Rv1 tumors from PET–DCL–DTX, PET–DCL–SH or DTX treated mice. Scale bar: 200 μm. * p < 0.05, ** p < 0.01, *** p < 0.001.

To examine the anti-cancer activity of PET–DCL–DTX *in vivo*, we established a xenografted PCa model by subcutaneously injecting 1 × 10^6^ PSMA-positive CWR22Rv1 cells into 6-week-old athymic male nude mice. Four weeks later, mice with tumors were randomly grouped and subjected to indicated administration by intraperitoneal (I.P) injection every other day: (i) drug-free PET–DCL–SH; (ii) drug-loaded PET–DCL–DTX and (iii) small molecule DTX. The therapeutic concentration of DTX in the nanoconjugate was 5 mg/kg. The tumor size and body weight were weekly monitored to examine the therapeutic efficacy and potential side effect of our therapies. The mice were sacrificed at the endpoint of the therapies and the tumors were dissociated for image capturing and weighing. As shown in Figure 9A–C, the CWR22Rv1 tumor growth was considerably constrained by PET–DCL–DTX and DTX administration, as compared to the drug-free carrier. Notably, one mortality (error symbol) and one tumor-free mouse (circle symbol) were observed during PET–DCL–DTX therapy. In contrast, DTX therapy led to three deaths (error symbol), suggesting that DTX is considerably more toxic than this nanodrug. Moreover, mice with PET–DCL–DTX administration exhibited improved health as compared to the DTX treated cohorts, as revealed by the alleviated body weight loss (Figure 9D). To further confirm the health benefits from PET–DCL–DTX therapy, we measured liver damage by histological and molecular analysis. Alanine aminotransferase (ALT) and aspartate transaminase (AST), two critical biomarkers of an injured liver, were obviously increased in the DTX treated groups as compared to the PET–DCL–DTX treated counterparts (Figure 9E). Additionally, as shown in Supplementary Figure S19, other biochemical indicators, including alkaline phosphatase (ALP) (liver injury biomarker), blood urea nitrogen (UREA) and creatinine (CREA) (renal toxicity biomarkers), also showed similar results. Consistently, the histologic analysis of the mice liver also indicated that the livers of the PET–DCL–DTX administrated mice had less infiltration of immune cells (red arrow) compared to those of the DTX treated controls (Figure 9F). Collectively, these data suggest that PET–DCL–DTX is functional *in vivo* to significantly suppress PSMA PCa tumor growth but has mitigated side effects.

It is intuitive to examine whether PET–DCL–DTX has an accumulation in the PCa tumors, thus exerting its anti-cancer effect. To this end, we labeled PET–DCL–DTX with a fluorescent dye (Cyanine 5, Cy5) and intraperitoneally administrated it into PCa bearing mice at an effective dose of 1 mg/kg. *In vivo* fluorescence imaging was conducted by the IVIS system to survey the dynamic distribution and targeting behavior over time. As shown in Supplementary Figure S20, the fluorescence signal appeared 1 h after administration, and its intensity throughout the body increased with time and reached the maximum within 8–12 h, then gradually decreased. It should be pointed out that the fluorescence intensity within the tumor was relatively prominent throughout the observation period, indicating that PET–DCL–DTX has excellent tumor targeting ability. Additionally, as exhibited in Figure 9G, after 24 h, the *ex vivo* fluorescent images showed that PET–DCL–DTX had a clear accumulation in PCa tumors as well as in liver organs. Above results suggest that PET–DCL–DTX can specifically recognize PSMA-positive PCa to suppress its growth.

Our aforementioned data also demonstrated that PET–DCL–DTX inhibited PCa cell growth at least partially by down-regulating the AR signaling and cell proliferation. To confirm these findings *in vivo*, we measured the expression levels of the AR and proliferation marker Ki67 in our xenografted mouse model by IHC staining. As shown in Figure 9H, PET–DCL–DTX as well as DTX significantly decreased the IHC score of AR and Ki67 compared to the drug-free vehicle, which is highly consistent with our *in vitro* data. These findings reinforced the anti-cancer activity of PET–DCL–DTX toward PSMA-positive PCa *in vivo*, which was largely due to its DTX loading capacity and the functional DCL group.

## Conclusion

In this study, we have designed and prepared a novel polymer–drug nanoconjugate (PET–DCL–DTX) derived from a PEG-based water-soluble polyester conjugating with anti-tumor drug (DTX) and the PSMA ligand (DCL) for treatment of PSMA-positive PCa. In the preparation of polyester, short-chain PEG was directly involved in polymerization as monomer, which not only overcame the problem of poor water solubility of polyester but also did not damage the drug-loading capacity (DTX content was ca. 7%). Benefitting from the ideal hydrophilic and hydrophobic balance, PET–DCL–DTX self-assembled into near-spheroids of about 57 nm in aqueous solution, and the hydrophobic DTX was camouflaged by the outer hydrophilic PEG and DCL, which facilitated the targeted delivery of large amounts of DTX to tumor and reduced the intrusion into normal tissues. The DCL enabled the nanoconjugate to have specific binding capacity to PSMA-positive prostate tumor cells, thus showing tumor subtype selectivity. After enter into tumor cells, the reduction-sensitive cleavage of the disulfide bonds caused DTX to break away from the polymer and reanimate. Additionally, compared with DTX, the nanoconjugate showed lower toxic side effects. In summary, we prepared a polyester synchronously possessing good water solubility and high drug-loading ability, and used it to construct a polymer–drug nanoconjugate with tumor tissue and subtype dual targeting for the treatment of PSMA-positive PCa. The nanoconjugate has shown high efficiency and safety both *in vitro* and *in vivo*, so it is a potential candidate for clinical application.

## Supplementary Material

rbag072_Supplementary_Data

## Data Availability

The raw data will be available on request.
