# Supplementary material for: A novel polyester–drug nanoconjugate with subtype selectivity for high efficacy against PSMA-positive prostate cancer
Source: Regen Biomater. 2026 Apr 11;13:rbag072. doi: 10.1093/rb/rbag072 (PMC13218377; doi:10.1093/rb/rbag072)
Supplement: rbag072_Supplementary_Data [file rbag072_supplementary_data.zip › 最终上传版-Supporting files-20260410.docx]

Supporting Information

A novel polyester-drug nanoconjugate with sub-type selectivity for high efficacy against PSMA-positive prostate cancer

Shiwei Guo^1,†^, Xiyan Xu^4,†^, Guobing Li^3,†^, Yongfeng Yang^1^, Zhihui Yan^2^, Xulin Wang^3^, Pei Jing^1^, Yong Zhou^3^, Man Jia^3^, Yuanfu Wang^2^, Yan Dai^1^, Siping Wei^1^, Ronghao Wang^3,^* Bo Cheng^2,^*

^1^Department of Pharmacy, the Affiliated Hospital of Southwest Medical University, Southwest Medical University, Luzhou, 646000, Sichuan, China

^2^Department of Urology, the Affiliated Hospital of Southwest Medical University, Luzhou, 646000, Sichuan, China

^3^Department of Biochemistry and Molecular Biology, School of Basic Medical Sciences, Southwest Medical University, Luzhou, 646000, Sichuan, China

^4^Department of Histology and Embryology, Southwest Medical University, Luzhou, 646000, Sichuan, China

* Corresponding address.

E-mail address: cb1949@swmu.edu.cn (B. Cheng), ronghao_wang@163.com (R. Wang).

^†^These authors contributed equally to this work.

Experimental procedures

Preparation of Mal-DCL-O^t^Bu [1]

Under N_2_ atmosphere, (S)-Di-Tert-Butyl 2-(3-((S)-6-Amino-1-(Tert-Butoxy)-1-Oxohexan-2-Yl)Ureido)Pentanedioate (DCL-O^t^Bu) (300 mg, 0.62 mmol), cat. Hydroquinone methyl ether (MEHQ) (4 mg, 0.031 mmol) and N,N-Diisopropylethylamine (DIEA) (321 mg, 2.48 mmol) were dissolved in dichloromethane (DCM) (3 mL), then to the solution was added drop-wise a solution of 3-Maleimidopropionic acid succinimidyl ester (Mal-NHS) (197 mg, 0.74 mmol) in DCM (2 mL) at ambient temperature. After addition, the reaction was continued to the end point (TLC confirmed). Then the reaction solution was cooled in ice bath and water (10 mL) was added tardily, two phase liquid was separated and the aqueous phase was extracted with DCM (10 mL*2), the combined DCM phase was washed by 1N HCl (10 mL*2) and saturated NaCl aqueous solution (10 mL*2) in turn. After being dried by anhydrous Na_2_SO_4_, the solvent was removed under vacuum concentration, and the residue was purified *via* pre-TLC (developing solvent: petroleum ethe/ethyl acetate=1:1.5) to obtain Mal-DCL-O^t^Bu as a white solid (280 mg, yield: 70.7%).

Preparation of Mal-DCL [1]

The solution of Mal-DCL-O^t^Bu (210 mg, 0.33 mmol) in DCM (2 mL) was cooled in ice bath, and CF_3_COOH (4 mL) was added tardily, controlling the dropping speed for keeping solution temperature less than 0 ℃. After addition, the reaction was continued to the end point (TLC confirmed). Then the solvent was removed under vacuum concentration, and the residue was washed with ether (10 mL*3) to obtain Mal-DCL as a white solid (120 mg, yield: 78.8%).

Preparation of SPDP-DTX [1]

Under N_2_, 3-(pyridin-2-yldisulfanyl)propanoic acid (SPDP-COOH) (300 mg, 1.39 mmol) was dissolved in DCM (10 mL), then dicyclohexylcarbodiimide (DCC) (575 mg, 2.78 mmol) and dimethylaminopyridine (DMAP) (170 mg, 1.39 mmol) were added, after full dissolution, Docetaxel (DTX) (1.1 g, 1.39 mmol) in DCM (10 mL) was added drop-wise, controlling the dropping speed for keeping solution temperature less than 0 ℃. Then the reaction was continued at ambient temperature to the end point (TLC confirmed), the reaction solution became turbid. Solids were filtered out, the solution was washed by 1N HCl (10 mL*3), H_2_O (10 mL*3) and saturated NaCl (10 mL*3). After being dried with anhydrous Na_2_SO_4_, the solvent was removed under vacuum concentration, and the residue was purified pre-TLC (developing solvent: PE/EA=1:1) to obtain SPDP-DTX as a white solid (847 mg, yield: 60.6%).

Preparation of diAC-NHBoc

Under N_2_ and the cooling of ice-salt bath, to a solution of N-Boc-serinol (5.0 g, 26.15 mmol) and Et_3_N (10.6 g, 104.75 mmol) in DCM (60 mL) was added drop-wise a solution of Acryloyl chloride (5.9 g, 65.19 mmol) in DCM (40 mL), controlling the dropping rate to maintain the system temperature below -4 ℃. After dropping, the reaction was maintained at -4 ℃ for 30 minutes, then naturally returned to room temperature, and the reaction was continued for 8 hours. TLC indicated the end of reaction. The solution was washed sequentially with 1N HCl (50 mL*3), water (50 mL*3) and brine (50 mL*3), then it was dried over anhydrous Na_2_SO_4_ and concentrated in vacuum to give a crude yellow solid which was purified by pre-TLC (petroleum ether/ethyl acetate (2:1) as eluent) to give diAC-NHBoc as a light-yellow solid (5.2 g, yield: 66.4%, purity: 99.4%)).^1^H NMR (400 MHz, *d_6_*-DMSO): δ 7.09-7.11 (d, *J*=8.0 Hz, 1 H), 6.33-6.38 (m, 2 H), 6.12-6.19 (m, 2 H), 5.95-5.98 (m, 2 H), 4.05-4.18 (m, 5 H), 1.37 (s, 9 H). ^13^C NMR (101 MHz, *d_6_*-DMSO) δ 165.39, 155.47, 132.04, 128.27, 78.36, 63.38, 48.42, 28.34. MS (ESI, *m*/*z*): 322.1 [M+Na]^+^, 200.2 [M+H-Boc]^+^, 244.1 [M+H-^t^Bu]^+^, 621.3 [2M+Na]^+^.

Preparation of diAC-STrt

First, diAC-NHBoc (1.8 g, 6.01 mmol) was dissolved in DCM (20 mL). Then under the cooling of ice bath, CF_3_COOH (20 mL) was added drop-wise to the above solution, controlling the dropping rate to maintain the system temperature below 4 ℃. After dropping, naturally returned to room temperature, and the reaction was continued for 4 hours, TLC indicated the end of reaction. The solution was concentrated in vacuum followed by vacuum drying to remove CF_3_COOH and DCM to give de-Boc mid-product as a light-yellow solid. Second, under N_2_ and the cooling of ice-salt bath, the de-Boc mid-product in N, N-Dimethylformamide (5 mL) was drop-wise added to a solution of Trt-COOH (2.5 g, 7.17 mmol), HBTU (3.4 g, 8.97 mmol), HOBt (1.5 g, 11.10 mmol) and DIEA (3.1 g, 23.99 mmol) in N, N-Dimethylformamide (20 mL), controlling the dropping rate to maintain the system temperature below 0 ℃. After dropping, naturally returned to room temperature, and the reaction was continued for 8 hours, TLC indicated the end of reaction. Under the cooling of ice-salt bath, to the reaction solution was added sat. NaHCO_3_ (150 mL) and ethyl acetate (150 mL). Organic layer and water layer were separated collection, the water layer was extracted with ethyl acetate (150 mL*2), and the organic layers were combined and washed successively with sat. NaHCO_3_ (150 mL*2), 1N HCl (150 mL*3) and brine (150 mL*3), then it was dried over anhydrous Na_2_SO_4_ and concentrated in vacuum to give a crude pale-white solid which could be recrystallized from methyl tert-butyl ether to obtain diAC-STrt as a white solid (2.3 g, yield: 72.2%, purity: 99.1%)).^1^H NMR (400 MHz, *d_6_*-DMSO): δ 8.06-8.08 (d, *J*=8.0 Hz, 1 H), 7.22-7.35 (m, 15 H), 6.30-6.34 (m, 2 H), 6.08-6.15 (m, 2 H), 5.90-5.93 (m, 2 H), 4.25-4.32 (m, 1 H), 4.07-4.17 (m, 4 H), 2.17-2.23 (m, 4 H). ^13^C NMR (101 MHz, *d_6_*-DMSO) δ 170.29, 165.17, 144.40, 131.90, 129.04, 127.99, 127.94, 126.68, 65.93, 62.82, 46.67, 33.95, 27.38. MS (ESI, *m*/*z*): 552.2 [M+Na]^+^.

Stability of PET-DCL-DTX in serum

The stability of PET-DCL-DTX was studied though detecting the change of the particle size and PDI as a function of time. PET-DCL-DTX was added in in different buffers (PBS, HEPES or 10%FBS in PBS), and then incubated at 37 ^o^C, and its sizes and PDI were measured via DLS at a pre-determined time.

Western blot

CWR22Rv1 cells were lysed in RIPA buffer and proteins (20 µg) were separated on 10% SDS/PAGE gel and then transferred onto Nylon membrane. After being blocked in 10% milk TBS buffer, membranes were incubated with appropriate dilutions of specific primary antibodies overnight at 4 ºC: AR (5153S, CST), PARP (9542, CST), GAPDH (14C10, CST). Next day, membranes were intensively washed and followed by incubation with secondary antibodies and visualized using with ODYSSEY CLX (LI-COR, USA).

MTT assay

1X10^4^ PCa cells were loaded into 24-well plates and treated with various concentration of PET-DCL-DTX, PET-DTX, the drug free carrier or DTX for 48 hours, and followed by incubation with 5 µg/ml 3-(4,5-Dimethylthiazolyl)-2,5-diphenyltetrazolium bromide (MTT, Sigma) for another 2 hours. MTT medium was discorded and cells were suspended by 500 µL DMSO. The absorbance at 570 nm was used as the MTT value.

Colony formation assay

CWR22Rv1 or PC3 cells (1X10^3^) were seeded into 6-well plates and exposed with 10 nM PET-DCL-DTX or the drug free carrier for 24 hours. Then drugs containing medium was replaced by fresh medium and cells were allowed to grow for two weeks, and followed by 0.1% crystal violet staining. Number of colonies was counted by image J software.

Flow cytometry

PCa cells with PET-DCL-DTX or drug free carrier treatment were collected and washed with cold PBS buffer, and fixed by 75% ethanol. After 30 mins incubation with annexin V (556547, BD) in PBS working buffer, cells were subjected to flow cytometry to determine the apoptotic rate.

Immunofluorescent staining (IF)

CWR22Rv1 cells on coverslips were incubated with or without 10 nM PET-DCL-DTX for 4 hours. Then cells washed with PBS three times and fixed in 4% paraformaldehyde for 15 mins, and followed by 0.3% Tixton 100 PBS treatment. After being blocked in 5% BSA/5% milk containing PBS at room temperature for 30 mins, cells were blotted with anti-PSMA antibody (1:100, D4S1F, CST) overnight at 4 ºC. Next day, cells were incubated with Alexa Fluor 488 (1:200, A11034, Invitrogen) for 1 hours at room temperature. 4',6-diamidino-2-phenylindole (DAPI) was utilized to stain the nuclei. Immunofluorescent images were captured by ZEISS confocal microscope.

Real time quantitative PCR

TRIzol reagent (TIANGEN BIOTECH CO.,LTD, Beijing) was used to extract RNA from PCa cells. 1 μg total RNAs were utilized to perform reverse transcription with ReverTra Ace™ qPCR RT Kit (TOYOBO, Japan). RT product was 20 -fold diluted and used as template to perform real time quantitative PCR in LightCycler 480 with QuantiNova SYBR Green dye (TOYOBO, Japan). GAPDH mRNA level was served as a normalizing control. The primers for PSA, TMPRSS2 and FKBP5 were mentioned as previously described.

H&E staining and Immunohistochemical staining (IHC)

PCa tumors or liver tissues from in xenografted mouse model were fixed in 10% (v/v) formaldehyde in PBS, embedded in paraffin and cut into 4 μm sections. The deparaffinized PCa tumor sections were treated with 3% peroxidase methanol for 15 min at room temperature to reduce the endogenous biotin background, and followed by recovery of masked epitopes in citrate buffer (pH=6.0). Tumor sections were then blocked by 5% BSA+5% milk PBS for 1 hour at room temperature and followed by incubation with anti-AR (1:100, 5153S, CST) or anti-Ki67 (1:200, 25229, CST) antibody at 4 ºC overnight. AR and Ki67 signals were visualized with DAB kit (Solarbio, Beijing). H&E staining was performed to liver tissue sections. Images were captured under microscope.

Supporting results and discussions

Stability of PET-DCL-DTX in serum

We have investigated the self-assembly behavior of PET-DCL-DTX in different buffers (PBS, HEPES or 10%FBS in PBS). The results (Figure S21) showed that the aqueous particle size and PDI in different buffers (PBS, HEPES or 10%FBS in PBS) have no obvious difference, indicating that PET-DCL-DTX can maintain a stable nanostructure in other more relevant physiological environments. Additionally, the stability of PET-DCL-DTX in mimic serum (10% FBS in PBS, pH 7.4) have also been investigated via DLS and the results (Figure S22) showed that within 48 hours of incubation, there were no significant changes in the particle size and dispersibility of PET-DCL-DTX, indicating its stability in serum.

Supporting Figures

**Figure S1. ^1^**H NMR spectrum of diAC-NHBoc (recorded in *d_6_*-DMSO).

**Figure S2. ^13^**C NMR spectrum of diAC-NHBoc (recorded in *d_6_*-DMSO).

**Figure S3.** LC-MS spectra of diAC-NHBoc, displaying a single ion peak at 322.1 m/z for [M+Na]^+^, 200.2 m/z for [M+H-Boc]^+^, 244.1 m/z for [M+H-^t^Bu]^+^, 621.3 m/z for [2M+Na]^+^.

**Figure S4.** MAIDI-HRMS spectrum of diAC-NHBoc, displaying a single ion peak at 322.1270 m/z for [M+Na]^+^.

**Figure S5.** HPLC spectrum of diAC-NHBoc. The purity is 99.4%.

**Figure S6. ^1^**H NMR spectrum of diAC-STrt (recorded in *d_6_*-DMSO).

**Figure S7. ^13^**C NMR spectrum of diAC-STrt (recorded in *d_6_*-DMSO).

**Figure S8.** LC-MS spectra of diAC-STrt, no molecular ion peak and only Trt fragment peak (243.2 m/z).

**Figure S9.** MAIDI-HRMS spectrum of diAC-STrt, displaying a single ion peak at 552.1825 m/z for [M+Na]^+^.

**Figure S10.** HPLC spectrum of diAC-STrt. The purity is 99.1%.

**Figure S11.** ^1^H NMR spectrum of PET-STrt (recorded in *d_6_*-DMSO).

**Figure S12.** GPC spectrum (A) and DLS analysis (B) of PET-STrt.

**Figure S13.** ^1^H NMR spectrum of PET-SH (recorded in *d_6_*-DMSO).

**Figure S14.** GPC spectrum (A) and DLS analysis (B) of of PET-SH.

**Figure S15.** ^1^H NMR spectrum of PET-DCL-SH (recorded in *d_6_*-DMSO).

**Figure S16.** GPC spectrum (A) and DLS analysis (B) of PET-DCL-SH.


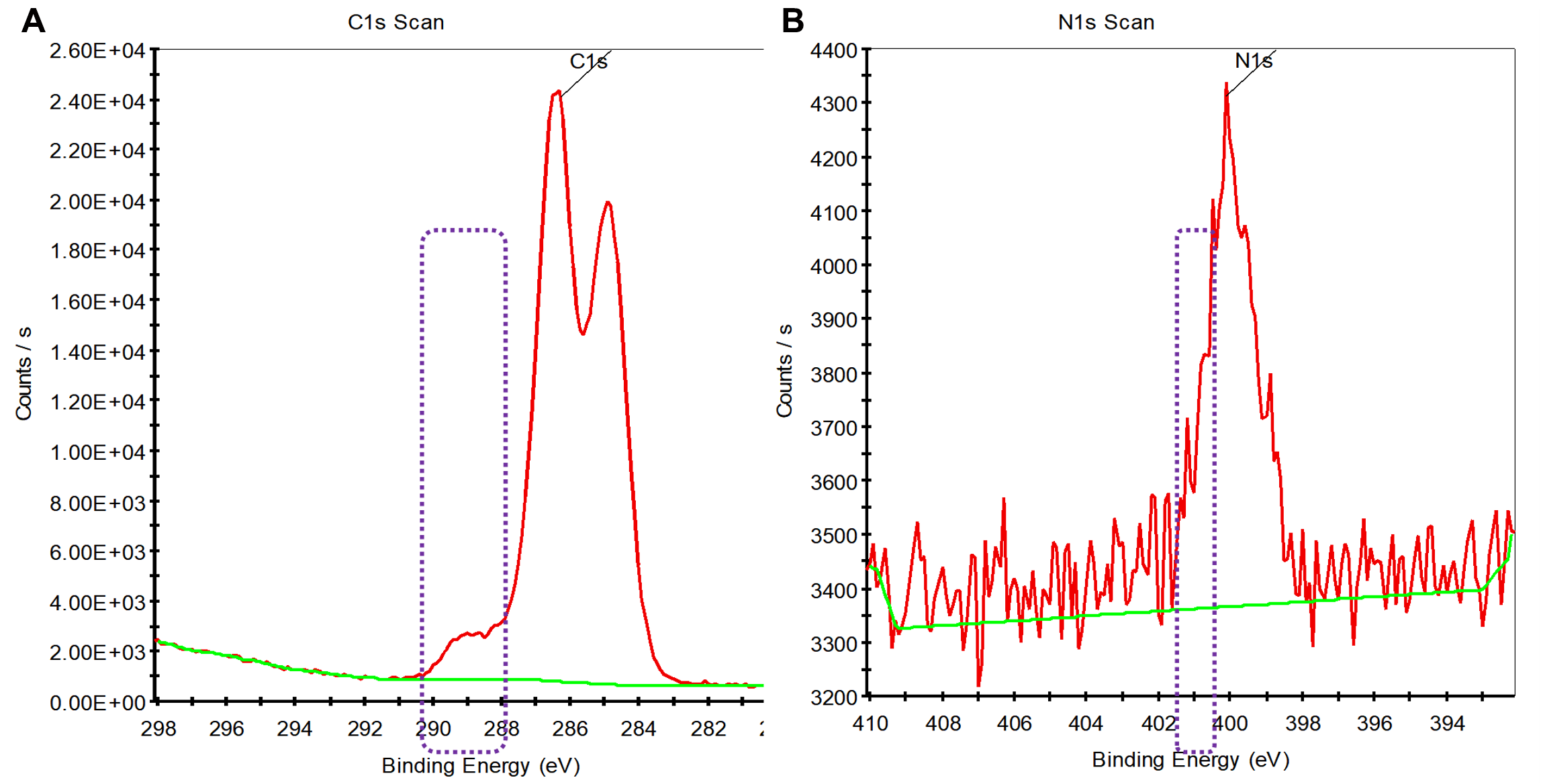


**Figure S17.** XPS spectra of PET-DCL-DTX.


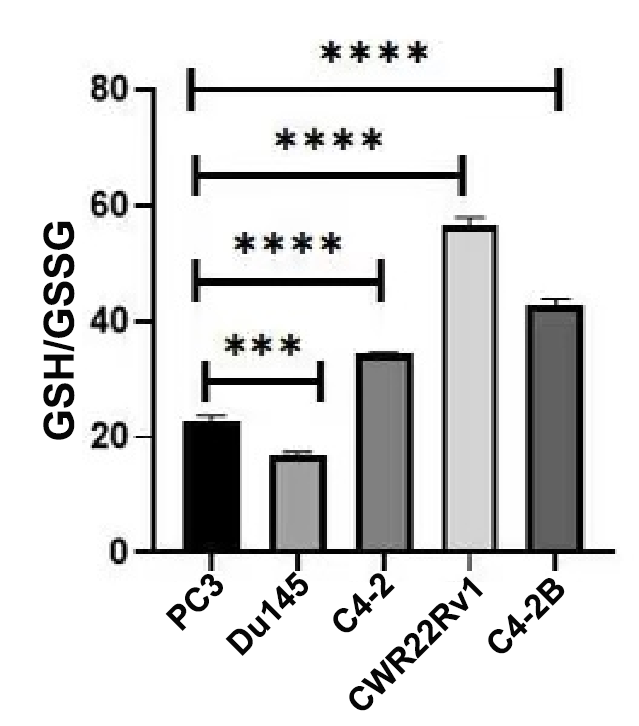


**Fig S18.** GSH/GSSG ratio in various PCa cells (Measured via zymography with OD412).

**Figure S19.** ALP, UREA and CREA levels in serum plasma from PET-DCL-DTX, PET-DCL-SH, or DTX treated mice.

**Figure S20.** In vivo fluorescence images of the mice bearing orthotopic PCa tumors after administration of Free Cy5 or PET-DCL-DTX.

**Figure S21.** Variations of aqueous particle size and PDI of PET-DCL-DTX at different buffers (PBS, HEPES or 10%FBS in PBS).

**Figure S22.** Stability of PET-DCL-DTX in serum (10% FBS in PBS, pH 7.4) (Measured *via* DLS).

References

1. Guo S, Cao Y, Cheng B, Zhou Y, Li X, Zhang M, et al. A nanoprodrug derived from branched poly (ethylene glycol) recognizes prostate-specific membrane antigen to precisely suppress prostate cancer progression. Int J Biol Macromol*.* 2024;282:136831.
